# Does Atherosclerosis Progress Faster After Liver Transplant? A Comparative Study and Analysis of Progression Risk Factors

**DOI:** 10.1155/joot/1851142

**Published:** 2026-08-18

**Authors:** Giulia Pagano, Judit Mestres-Martí, Cautar El Maimouni, Clara Viñals, Jose T. Ortiz-Pérez, Pablo Ruiz, Jordi Colmenero, Emilio Ortega, Gonzalo Crespo

**Affiliations:** ^1^ Liver Transplant Unit, Hospital Clínic, Barcelona, Spain, hospitalclinic.org; ^2^ Department of Hepatology, Hospital Clínic, Barcelona, Spain, hospitalclinic.org; ^3^ University of Barcelona, Barcelona, Spain, ub.edu; ^4^ Department of Radiology, Hospital Clínic, Barcelona, Spain, hospitalclinic.org; ^5^ Department of Endocrinology and Nutrition, Hospital Clínic, Barcelona, Spain, hospitalclinic.org; ^6^ Department of Cardiology, Hospital Clínic, Barcelona, Spain, hospitalclinic.org; ^7^ IDIBAPS, Barcelona, Spain, idibaps.org; ^8^ CIBER-EHD, Madrid, Spain, ciberehd.org; ^9^ CIBER-OBN, Madrid, Spain

**Keywords:** cardiovascular disease, cirrhosis, coronary calcium calcification score, immunosuppression, long-term complications

## Abstract

**Background:**

Cardiovascular disease (CVD) is thought to evolve faster after liver transplantation (LT) due to an increased prevalence of cardiovascular risk (CVR) factors and the use of immunosuppression. The progression of coronary artery calcium score (CACS) in computed tomography is a prognostic biomarker of CVD. There is scarce information about CACS changes after LT or in patients with cirrhosis.

**Methods:**

We compared the 5‐year progression of CACS in two populations: 75 LT recipients and 20 patients with cirrhosis assessed for LT but who eventually did not undergo LT (non‐LT). In both populations, CACS was measured at LT evaluation and 5 years thereafter. Additionally, we studied 1‐year post‐LT CVR factors and immunosuppression parameters, including cumulative exposure to tacrolimus (CET) as potential predictors of 5‐year CACS progression after LT.

**Results:**

Both groups were comparable regarding CVR factors, time between CACS, or baseline CACS. CACS increased in both groups, with an annualized progression of 23 Agatston units (AU)/year in LT and 30 AU/year in non‐LT (*p* = 0.833). CVR factors control after LT was optimal, and 80% received standard/minimized CET. Higher pre‐LT CACS (*p* < 0.001) and worse kidney function 12 months after transplantation (*p* = 0.003) independently predicted progression after LT. No immunosuppression‐related variables were associated with CACS progression.

**Conclusion:**

In LT recipients with optimized control of CVR factors and standard/minimized CET, CACS progression was not faster than in patients with cirrhosis. The association between posttransplant renal function and CACS progression warrants further investigation.

## 1. Introduction

Cardiovascular disease (CVD) is an important cause of death after liver transplantation (LT) [1–4]. Despite improvements in surgical techniques and posttransplant care, long‐term mortality in LT recipients is increasingly driven by cardiovascular complications rather than graft‐related causes. LT recipients are thought to be more susceptible to CVD than the general population due to an increasing prevalence of steatotic liver diseases (SLD) and cardiometabolic comorbidities, coupled with an older age of recipients [5, 6]. In addition to traditional cardiovascular risk (CVR) factors, LT recipients are exposed to transplant‐specific mechanisms that may accelerate atherosclerosis. Immunosuppressive therapy has been consistently associated with the development of post‐LT metabolic syndrome, including hypertension, diabetes mellitus, and dyslipidemia. Consequently, immunosuppression is considered to impact the risk of posttransplant CVD, although its precise role and relative contribution remain poorly defined [7]. It is also unknown whether the risk of post‐LT CVD is indeed higher than that of non‐LT patients with cirrhosis.

Coronary artery calcium score (CACS) in computed tomography (CT) is a biomarker of atherosclerosis that predicts cardiovascular events (CVEs) and mortality in the general population and in LT recipients [8–13]. The progression of CACS over time also predicts CVD and mortality in the general population [14–18]. Therefore, assessing CACS progression may provide incremental value for CVR stratification beyond traditional risk factors. The identification of individuals with accelerated CACS progression could represent a useful strategy to recognize patients who may benefit more from preventive interventions. Furthermore, studying CACS progression may help to better understand the mechanisms leading to CVD after LT. However, data on the progression of coronary atherosclerosis in LT recipients are scarce. To date, only one study has assessed the progression of coronary atherosclerosis in a small cohort of 30 LT recipients [19]. This significant knowledge gap limits our ability to define the true burden and determinants of atherosclerosis progression in this population, and to tailor appropriate prevention strategies.

Consequently, we conducted this study with two main aims: to analyze the progression of CACS in LT recipients and compare it with that of patients with cirrhosis and to identify the factors associated with this progression after LT, with a particular focus on the burden of CVR factors and immunosuppressive therapy during the first post‐LT year.

## 2. Materials and Methods

### 2.1. Study Design and Population

We conducted a single‐center, cross‐sectional study in which we considered all LT recipients that underwent LT evaluation, including CACS as part of pre‐LT cardiac assessment between 2017 and 2020 (CACS_bl_) at the Liver Transplant Unit of Hospital Clinic, Barcelona. In addition, we identified those patients who had been evaluated for LT including a CACS_bl_ as part of pre‐LT assessment during the same time period and who were eventually not transplanted due to recompensation (non‐LT). The indication of coronary CT with CACS in the pre‐LT assessment of candidates was protocolized and based in the presence of CVR factors [20]. During pre‐LT cardiovascular assessment, high‐risk cardiovascular patients were not systematically excluded from LT solely on the basis of CVR factors or elevated CACS. At our center, candidates were considered unsuitable for transplantation only in the presence of nonrevascularized severe coronary artery disease, defined as stenosis > 70% in a major epicardial coronary artery or in a branch supplying a significant myocardial territory, or in the presence of untreatable severe valvular heart disease or heart failure. These decisions were made after multidisciplinary evaluation involving hepatologists, cardiologists, anesthesiologists, and transplant surgeons. Between 2022 and 2024, approximately 5 years after CACS_bl_, patients were contacted to perform a follow‐up CACS (CACS_fu_).

Inclusion criteria were as follows: (1) evaluation for LT between 2017 and 2020, with the outcome of evaluation being either LT or no indication of LT due to recompensation, and (2) baseline coronary CT with CACS_bl_ performed as part of the pre‐LT cardiac assessment. Exclusion criteria included cardiac procedures performed between CACS_bl_ and the cross‐sectional assessment including coronary stent implantation and cardiac surgery, regardless of whether these occurred before or after LT.

The primary endpoint was CACS progression and its comparison between LT recipients and non‐LT patients. Secondary endpoints included the identification of factors associated with CACS progression after LT, focusing on the CVR factor burden and immunosuppressive therapy during the first post‐LT year.

### 2.2. CACS Calculation and Definition of CACS Progression

The imaging protocol consisted of a multidetector CT scan acquired in the axial plane with electrocardiographic gating. Acquisition parameters included a slice thickness of 3 mm, and an interslice interval of 1.5 mm. Image reconstruction was performed using a 3‐mm slice thickness in a soft tissue kernel, displayed in the axial view. CACS was calculated by a semiautomated computerized software program using the Agatston method [21], expressed as Agatston units (AU) in absolute numbers and categorized in (I) the absence of (CACS = 0), and (II) mild (CACS 1–99), (III) moderate (100–399), or (IV) severe calcified atherosclerosis (CACS ≥ 400).

Any progression of CACS was defined as any increase between CACS_bl_ and CACS_fu_. Progression was quantified using the annualized difference between follow‐up and baseline CACS (CACS_fu_–CACS_bl_)/time between scans in years), a method that has been shown to predict mortality and CVEs [13, 15, 16, 18]. CACS progression was analyzed in the whole populations of LT vs. non‐LT and also differentiating patients with (CACS_bl_ > 0) and without (CACS_bl_ = 0) prevalent coronary calcifications [14].

### 2.3. Variables Collected and Patient Follow‐Up

Baseline information was obtained from all participants. CVR factors were defined according to the International Classification of Diseases–11 codes. Metabolic syndrome was diagnosed according to the established criteria [22].

Follow‐up of LT recipients in our center has been previously described [23]. The prevalence and degree of control of CVR factors and renal function 1 year after LT were prospectively recorded. Immunosuppression during the first year after LT was documented retrospectively, including cumulative dose of steroids during the first post‐LT year and cumulative exposure to tacrolimus (CET) 3 and 12 months after LT. CET was calculated according to the method proposed by Rodríguez‐Perálvarez et al. [24]. Thresholds for high exposure, conventional exposure, minimization, and aggressive minimization have been previously defined [24].

Nontransplanted patients were followed according to the standard clinical practice by their referral hepatologists. Data regarding CVR factors 12 months after CACS_bl_ were retrieved retrospectively.

For both groups, we recorded the incidence of CVE, during the period of time between CACS_bl_ and CACS_fu_, including acute coronary syndrome comprehending myocardial infarction with or without ST elevation or unstable angina, ischemic or hemorrhagic stroke, arrhythmia (ventricular tachycardia, atrial flutter, or fibrillation), peripheral artery disease, and heart failure.

### 2.4. Sample Size Calculation

In the only study on CACS progression in LT recipients [19], any progression in CACS after a median follow‐up of 4.5 years was seen in 70% of patients. Considering this, a sample size of 75 patients would be necessary to estimate any progression of CACS in our population of LT recipients with pre‐LT CACS with 95% confidence and 5% precision, and assuming 15% of losses. With respect to nontransplanted patients, we used a convenience sample size considering the absence of data in this population and the small population of LT candidates with CACS_bl_ that are not admitted in the waiting list after recompensation.

### 2.5. Statistical Analysis

Categorical variables are presented as absolute numbers and percentages, whereas quantitative variables are summarized as medians and interquartile ranges (IQR), given their nonnormal distribution and/or the limited sample size. The statistical evaluations used in this study included comparative analyses for categorical and continuous variables, paired analyses, and regression modeling. Comparisons of categorical variables were performed using Fisher’s exact test. Comparisons of continuous variables between two independent groups were assessed using the Mann–Whitney *U* test, whereas comparisons among more than two independent groups were performed using the Kruskal–Wallis test. For paired data, McNemar’s test was used for categorical variables, and the Wilcoxon signed‐rank test was used for continuous variables.

The association between the annualized progression of CACS and clinical variables assessed 1 year after LT was evaluated using univariable and multivariable linear regression analyses. Variables showing an association with annualized CACS progression at a *p* value < 0.10 in the univariable analysis were entered into the multivariable linear regression model. Regression results were reported as regression coefficients with corresponding confidence intervals and *p* values, when applicable.

All statistical tests were two‐sided, and a *p* value < 0.05 was considered statistically significant. All statistical analyses were performed using IBM SPSS Statistics for Windows, Version 28.0 (IBM Corp., Armonk, NY, USA)

## 3. Ethical Issues

The study was performed in accordance with the Declaration of Helsinki and approved by the Clinical Research Ethical Committee of Hospital Clínic (HCB/2022/0580). All patients provided written informed consent before being included.

## 4. Results

### 4.1. Patients

**FIGURE 1:**
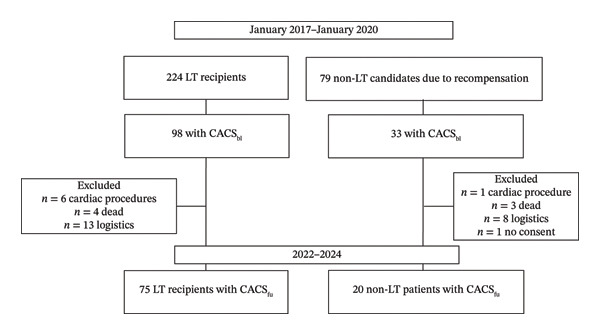
Flow diagram of the study. CACS, coronary artery calcium score; LT, liver transplantation.

**FIGURE 2:**
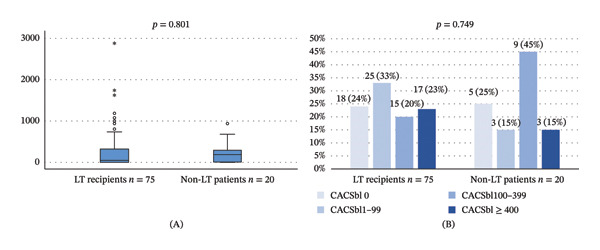
Baseline CACS in LT and non‐LT patients. (A) Box plots of CACS_bl_ in both groups; (B) CACS_bl_ categorized in CACS = 0, 1–99, 100–399, and ≥ 400 Agatston units.

Seventy‐five LT recipients and 20 non‐LT patients were included (Figure 1). Baseline characteristics, which were similar between the groups, are shown in Table 1. There were no differences in median CACS_bl_, which was 42 (0–2896) in LT and 107 (0–937) in non‐LT groups (*p* = 0.801) (Figure 2A). Categorization of CACS_bl_ was also similar (*p* = 0.749) (Figure 2B).

**TABLE 1:** Baseline characteristics of patients included.

Variable	LT recipients (*n* = 75)	Non‐LT patients (*n* = 20)	*p* value
Sex (male)	58 (77%)	17 (85%)	0.551
Age at baseline, years	60 (44–69)	58 (33–65)	0.066
Etiology (nonmutually exclusive)			
HCV	16 (21%)	5 (25%)	0.765
Alcohol	37 (49%)	10 (50%)	1.000
MASLD	30 (40%)	7 (35%)	0.799
Indication			
Decompensated cirrhosis	47 (63%)	15 (75%)	0.304
Hepatocellular carcinoma (HCC)	28 (37%)	5 (25%)	0.429
Body mass index ≥ 30 kg/m^2^	36 (48%)	9 (45%)	1.000
Diabetes	35 (47%)	8 (40%)	0.624
Arterial hypertension	28 (37%)	6 (30%)	0.609
Dyslipidemia	16 (21%)	7 (35%)	0.243
Smoking habit			0.682
Past	35 (48%)	13 (65%)	
Active	11 (15%)	2 (10%)	
HIV	4 (5%)	2 (10%)	0.603
CACS_bl_	42 (0–2896)	107 (0–937)	0.801
Months between CACS_bl_ and LT	5.2 (2.5–9)	N/A	N/A
Months between CACS_bl_ and CACS_fu_	64 (57–71)	58 (48–70)	0.131

*Note:* Data are given as absolute count (%) or median (IQR). Differences were assessed with the Fisher test for categorical variables and the Mann–Whitney/Kruskal–Wallis tests for continuous variables. MASLD, metabolic dysfunction‐associated steatotic liver disease.

Abbreviations: CACS, coronary artery calcification score; HCV, hepatitis C virus; HIV, human immunodeficiency virus; LT, liver transplantation; N/A, nonapplicable.

### 4.2. CACS Progression

**FIGURE 3:**
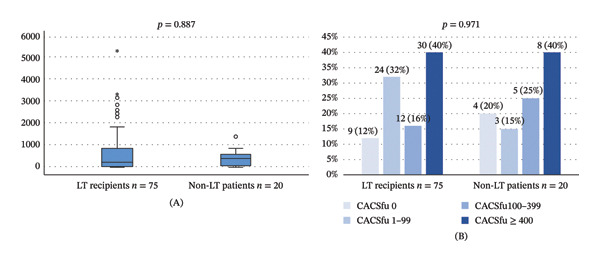
Follow‐up CACS in LT and non‐LT patients. (A) Box plots of CACS_fu_ in both groups; (B) CACS_fu_ categorized in CACS = 0, 1–99, 100–399, and ≥ 400 Agatston units.

**FIGURE 4:**
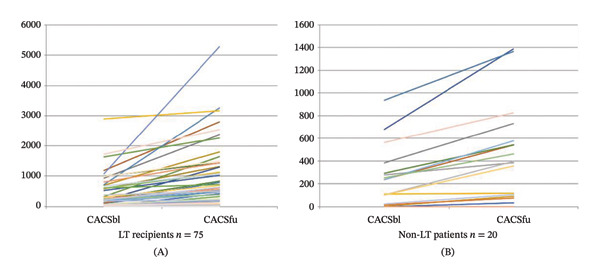
Individual changes in CACS (CACS_bl_ to CACS_fu_) in LT recipients (A) and non‐LT patients (B).

CACS_fu_ was performed at a median time of 64 and 58 months after CACS_bl_ in LT and non‐LT patients, respectively (*p* = 0.131), with a median time of 5 months between CACS_bl_ and LT in the LT group. Median CACS_fu_ in each group was 208 (28–841) and 371 (45–570), respectively (*p* = 0.887) (Figure 3A), and categorization of CACS_fu_ is shown in Figure 3B. Any progression of CACS with respect to CACS_bl_ was seen in 85% and 80% of LT and non‐LT patients (*p* = 0.512). Median annualized progression of CACS was 23/year (2–86) in LT recipients and 30/year (4–57) in non‐LT (*p* = 0.833). The change between CACS_bl_ and CACS_fu_ for each individual patient in both groups is shown in Figure 4.

We also performed an exploratory subgroup analysis, stratifying patients according to the presence or absence of diabetes. Among diabetic patients, no statistically significant differences were observed between LT and non‐LT patients in CACS_bl_ (*p* = 0.490), CACS_fu_ (*p* = 0.939), or annualized CACS progression (*p* = 0.725). Similarly, among nondiabetic patients, no significant differences were found in CACS_bl_ (*p* = 0.810), CACS_fu_ (*p* = 0.957), or annualized progression (*p* = 0.905).

**FIGURE 5:**
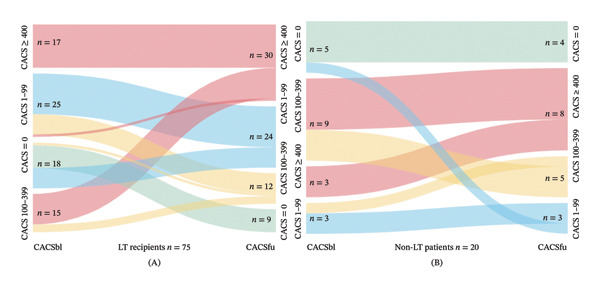
Sankey plot showing the flow of patients between the different categories of CACS between baseline and follow‐up in the LT (A) and non‐LT (B) cohorts.

We then analyzed in separate patients with CACS_bl_ = 0 and those with CACS_bl_ > 0. For patients with CACS_bl_ = 0 (*n* = 18 LT, *n* = 5 non‐LT), any incident CACS_fu_ was seen in 9/18 (50%) LT and 1/5 (20%) non‐LT, *p* = 0.339. In the 9 LT progressors with CACS_bl_ = 0, median annualized progression was 2 (1–7), while in the only non‐LT progressor with CACS_bl_ = 0, annualized progression was 10 (*p* = 0.4). For patients with CACS_bl_ > 0 (*n* = 57 LT, *n* = 15 non‐LT), any progression was seen in 55/57 LT (96%) and 15/15 (100%) non‐LT, *p* = 1. In the LT and non‐LT patients with CACS_bl_ > 0 and progression, median annualized progression was, respectively, 40 (8–102) and 45 (18–66), *p* = 0.830. Changes in the categories of CACS in both groups are shown in Figure 5A,B.

### 4.3. CVR Factors and Immunosuppression 12 months After LT

CVR factors before and 12 months post‐LT in the LT cohort are shown in Table 2. The prevalence of CVR factors significantly increased after LT, as did the use of treatment for these conditions.

**TABLE 2:** Cardiovascular risks factors before and 12 months after liver transplant (*n* = 75).

Variable	Before LT	12 months post‐LT	*p* value
Arterial hypertension	28 (37%)	38 (51%)	0.034
Systolic blood pressure (mmHg)	121 (110–137)	129 (120–141)	0.028
Diastolic blood pressure (mmHg)	69 (62–78)	77 (71–81)	< 0.001
Treatment for arterial hypertension	13 (17%)	32 (43%)	< 0.001
Blood pressure < 140/90 mmHg	59 (78%)	55 (73%)	0.719
Dyslipidemia	16 (21%)	42 (56%)	< 0.001
Total cholesterol (mg/dL)	147 (130–180)	178 (159–203)	0.341
LDL‐C (mg/dL)	94 (73–128) (*n* = 26)	109 (87–120)	< 0.001
HDL‐C (mg/dL)	49 (33–63) (*n* = 26)	46 (36–54)	0.837
Triglycerides (mg/dL)	77 (61–102)	128 (93–191)	< 0.001
Treatment for dyslipidemia	5 (7%)	25 (33%)	< 0.001
Diabetes	35 (47%)	49 (65%)	0.001
HbA1C (%)	6.2 (5.2–6.9) (*n* = 28)	5.8 (5.2–6.4)	0.957
HbA1C < 7%	22 (78%) (*n* = 28)	65 (87%)	0.257
Treatment for diabetes	30 (40%)	43 (57%)	0.011
Body mass index (kg/m^2^)	29.7 (26–32.8)	28.4 (25.7–32.9)	0.303
Body mass index ≥ 30 kg/m^2^	36 (48%)	28 (41%)	0.227
eGFR (mL/min/1.73 m^2^)	71 (51–90)	61 (47–75)	< 0.001
Active smoking	11 (15%)	6 (8%)	0.125

*Note:* Data are given as absolute count (%) or median (IQR). HbA1c, glycosylated hemoglobin. Comparisons were performed using the Wilcoxon test.

Abbreviations: eGFR, estimated glomerular filtration rate; HDL‐C, high‐density lipoprotein cholesterol; LDL‐C, low‐density lipoprotein cholesterol.

One year after LT, the main immunosuppressor was tacrolimus in 65 patients (87%), cyclosporine A in 6 (8%), and everolimus in 4 (5%). Most patients received combination therapy with mycophenolate: 58 (77%) at Month 1, 42 (56%) at Month 3, 39 (52%) at Month 6, and 32 (43%) at Month 12 post‐LT, and 10 patients had everolimus in combination with tacrolimus. Median cumulative steroid dose during the first 12 months after LT was 3608 mg (1455–13815). CET at 90 days corresponded to aggressive minimization in 9% (*n* = 6) of patients, minimization in 28% (*n* = 18), conventional in 54% (*n* = 35), and high in 9% (*n* = 6), while figures for the same categories at CET 12 months were 6% (*n* = 4), 22% (*n* = 14), 52% (*n* = 34), and 20% (*n* = 13), respectively.

### 4.4. CVR Factors 12 months After CACS_bl_ in Non‐LT Patients

Among the 20 non‐LT patients, one year after CACS_bl_, 4 (20%), 8 (40%), and 2 (10%) received blood pressure (BP)–lowering, antidiabetic, and lipid‐lowering treatment. BP measurement was available in 15 patients, being 130 (124–136)/78 (70–81) mmHg, with 12/15 (80%) patients having BP < 140/90 mmHg. Median HbA1c, available in 11 patients, was 5.4% (4.8–7.9), with 7/11 (64%) having HbA1c < 7%. Regarding lipids, only 5 patients had LDL‐cholesterol measurements, whose median values were 88 mg/dL (42–117), while total cholesterol and triglyceride levels, measured in the whole cohort, were, respectively, 180 (140–191) and 92 (62–132) mg/dL.

### 4.5. Variables Associated With CACS Progression in LT Recipients

Uni‐ and multivariable linear regression analyses of variables associated with CACS progression after LT are shown in Table 3. At univariable analysis, older age, higher CACS_bl_, and diabetes and eGFR 1 year after LT were statistically associated with CACS progression. There was no association between CACS progression and CET or cumulative steroid dose. On multivariable analysis, a lower eGFR 12 months after LT and higher CACS_bl_ were independent predictors of an increased annualized progression of CACS.

**TABLE 3:** Uni‐ and multivariate linear regression analyses of variables associated with the annualized progression of CACS.

Variable	Univariate			Multivariate		
	B	95% CI	*p* value	B	95% CI	*p* value
Age, years	4.047	0.074, 8.021	0.046	1.588	−1.951, 5.127	0.374
Sex (male)	15.446	−27.197, 58.089	0.457			
CACS_bl_	0.103	0.071, 0.136	< 0.005	0.102	0.061, 0.142	< 0.001
SBP^∗^	0.440	−0.972, 1.852	0.536			
DBP^∗^	1.016	−1.297, 3.329	0.384			
BP < 140/90 mmHg^∗^	−20.915	−71.794, 29.964	0.415			
DM^∗^	49.131	4.586, 93.675	0.031	26.281	−12.44, 65.001	0.180
HbA1c^∗^	16.144	−3.818, 36.105	0.111			
HbA1C < 7%^∗^	−19,025	−91.428, 53.378	0.602			
Total cholesterol^∗^	0.134	−0.522, 0.791	0.684			
LDL‐C^∗^	0.918	−0.159, 1.995	0.093			
HDL‐C^∗^	−0.362	−2.680, 1.957	0.755			
Triglycerides^∗^	0.045	2.144, 104.207	0.772			
Use of statins^∗^	43.518	−2.568, 89.605	0.064	−20.721	−61.127, 19.685	0.310
Metabolic syndrome^∗^	8.345	−35.961, 52.652	0.708			
eGFR^∗^	−1680	−2.890, −0.470	0.007	−1583	−2.611, −0.555	0.003
Active smoking^∗^	−15,929	−97.853,65.994	0.699			
Cumulative steroid^∗^	0.003	−0.012, 0.017	0.733			
Use of cyclosporine^∗^	50.270	−20.284, 120.823	0.160			
Use of mycophenolate^∗^	−5.441	−50.060, 39,179	0.809			
Use of everolimus^∗^	47.282	−8.296, 102.860	0.094	−4.101	−53.624, 45.423	0.869
CET 3 m post‐LT	−0.043	−0.171, 0.084	0.500			
CET 12 m post‐LT	−0.023	−0.053, 0.007	0.125			

*Note:* HbA1c, glycosylated hemoglobin; considering collinearity between LDL‐C and use of statins, we included the latter in the multivariate analysis.

Abbreviations: CACS, coronary artery calcification score; CET, cumulative exposure to tacrolimus; DBP diastolic blood pressure; DM, diabetes mellitus; eGFR, estimated glomerular filtration rate; HDL‐C, high‐density lipoprotein cholesterol; LDL‐C, low‐density lipoprotein cholesterol; SBP, systolic blood pressure.

^∗^Variables 12 months post–liver transplant.

### 4.6. CVEs

In the interscan period, 7/75 (9%) and 2/20 (10%) LT and non‐LT patients, respectively, presented a CVE (*p* = 1). In the LT group, all events took place after LT, and there were no statistically significant differences in the type of CVE between the groups (Table 4). LT recipients who presented a CVE had a median annualized progression of CACS of 247 (25–389), while recipients who did not present a CVE showed a median annualized progression of 17 (2–79) (*p* = 0.018). The OR of the annualized progression of CACS to predict CVE in the interscan period was 1.014 (95% CI 1.004–1.023), *p* = 0.004. When controlled by CACS_bl_, CACS progression retained statistically significant association with CVE (OR 1.015, 95% CI 1.003–1.026, *p* = 0.003), while CACS_bl_ was not associated with CVE (OR 1.001, 95% CI 0.997–1.002, *p* = 0.773).

**TABLE 4:** Interscan CVEs for LT recipients and non‐LT patients.

Interscan CVEs	LT recipients (*n* = 75)	Non‐LT patients (*n* = 20)	*p* value
Number of CVEs	7 (9%)	2 (10%)	1
Distribution of events			0.384
Acute coronary syndrome^∗^	0	0	
Ischemic or hemorrhagic stroke	1 (14%)	1 (50%)	
Arrhythmia^∗∗^	3 (43%)	1 (50%)	
Peripheral artery disease	1 (14%)	0	
Heart failure	2 (29%)	0	

*Note:* CVE, cardiovascular events.

Abbreviation: LT, liver transplantation.

^∗^Including myocardial infarction with or without ST elevation or unstable angina.

^∗∗^Including ventricular tachycardia, atrial flutter, or fibrillation.

## 5. Discussion

We analyzed CACS progression after 5 years in LT recipients and patients with cirrhosis that eventually did not need LT. Despite the notion that LT is associated with an accelerated progression of atherosclerosis, both groups presented a similar CACS progression. The fact that both groups had a similar incidence of interscan CVE is in line with the absence of differences in CACS progression. The association between CACS progression and CVE was independent from CACS_bl_, suggesting that CACS progression may be a surrogate for CVD progression after LT.

There may be different explanations for our results. First, the velocity of progression of atherosclerosis in patients with cirrhosis is unknown. In these patients, liver disease is the most important cause of death and consequently the risk of progression of CVD has been scarcely investigated and is difficult to study. Some data suggest that increased noninvasive markers of liver fibrosis are associated with an increased prevalence of coronary stenosis and carotid atherosclerosis in patients with SLD [25]. Indeed, cirrhosis is a proinflammatory state, and inflammation plays a fundamental role in all stages of CVD [26], and it has been postulated that advanced liver fibrosis may be partially associated with cardiovascular death through immune activation [27]. In addition, our patients underwent CACS_bl_ due to the clinical risk of silent CAD after assessment of CVR factors; consequently, their baseline risk of significant and progressive CVD was not negligible. According to our results, the progression of CVR in patients with cirrhosis and metabolic comorbidities may be higher than expected. Importantly, the follow‐up of CVR factors of the non‐LT cohort was less intense than that of LT recipients, with much lower use of BP‐lowering, antidiabetic, and lipid‐lowering treatment 12 months after CACS_bl_ than the LT cohort. In this population, it is difficult to estimate CVR due to the pathophysiological changes inherent to liver disease. Despite their “cardiovascularly benign” features, the progression of CACS was similar to a cohort of LT recipients with a higher incidence of CVR factors, who were treated more intensively.

Indeed, another potential explanation for the absence of differences could be related to an alleged mitigated progression of atherosclerosis in the LT group. The increase in the components of metabolic syndrome after LT [28] was also shown in our cohort. Despite this, post‐LT CVR factors were overall well controlled in our cohort: 87% of patients had HbA1c < 7%, 73% had BP < 140/90 mmHg, and 1/3 of patients were on lipid‐lowering treatment, all of which are higher figures than previous reports [29–31]. Previous studies have shown the barriers faced when trying to improve cardiovascular care of LT recipients [32]. In our center, a multidisciplinary strategy, coled by transplant hepatologists, endocrinologists, and advanced practice nurses and using protocol‐defined targets, resulted in a decrease in post‐LT CVE [23], and this may also be associated with a slower progression of CACS. This is consistent with the data showing that optimized BP during the first year after LT is associated with outcomes [31]. Prospective studies with larger sample size would be necessary to confirm this hypothesis.

While immunosuppressants worsen the cardiovascular profile of LT recipients [7], neither CET nor exposure to steroids nor other immunosuppressants 1 year after LT was associated with CACS progression. Only CACS_bl_ and kidney dysfunction were independently associated with CACS progression. CACS_bl_ is the main predictor of CACS progression in the general population [14] and also in the only previous experience in LT recipients [19]. It has been shown that in patients with chronic kidney disease, impaired renal function accelerates the progression of CACS, and, similarly, there is an association between high CACS and the progression of kidney failure [33–35]. In LT, a steady decrease in the median eGFR after LT due to calcineurin‐inhibitors exposure and to the cardiometabolic risk factors has been described [36]. Moreover, the trajectory of kidney function in the first year after LT has been associated with the incidence of CVE [37, 38], and creatinine levels 1 year after LT have also been shown to predict CVE [39]. Chronic kidney disease is a multifactorial condition closely linked to an increased risk of CVE and cardiovascular mortality [40]. Considering kidney dysfunction as a downstream consequence of CVR factors [41], it is plausible that not the individual effect of these factors but their convergent combination in renal function be the driver of CACS progression in our cohort. This possibly also partially explains the lack of impact of CET in CACS progression, together with the fact that overexposure to tacrolimus was infrequent in our cohort. However, given the observational design of the study, causality between kidney dysfunction and CACS progression cannot be established. Preserved kidney function may represent a marker of overall cardiovascular health rather than a direct determinant of slower atherosclerotic progression. Therefore, this finding should be considered hypothesis‐generating and warrants confirmation in prospective studies specifically designed to evaluate mechanistic relationships between renal function and coronary artery calcification.

A significant strength of our study is the inclusion of a control cohort, an uncommon feature in this kind of studies. Our controls were patients with cirrhosis that eventually recompensated, and whose baseline characteristics and CVR factors were similar to the study group. This is a valuable and difficult to retrieve group of patients in whom nonhepatic outcomes are understudied. We did not compare CACS progression with a comparable sample of the general population, which would be highly challenging to identity in terms of comparable baseline characteristics, but can frame our results within the data published in other studies. As an example, in the MESA study [14], for those with prevalent CACS (CACS_bl_ > 0), the median progression was 28.9/year, which seems somehow lower than 40 and 45 in our LT and non‐LT cohorts. Possibly more striking, only 15% of patients with CACS_bl_ = 0 progressed in the MESA study, as compared with 50% of our LT cohort. There were only 5 non‐LT patients with CACS_bl_ = 0, and progression was seen in 1 of them (20%). In a previous study in LT recipients [19], median CACS progression was 5 (13.7 in patients with baseline CACS > 0), much lower than that in our study. These differences probably respond to the different sample sizes and the characteristics (including CACS_bl_) of the cohorts included.

We acknowledge several limitations in our study. First, the small sample size of the non‐LT group compared with the LT cohort. Given such sample size, comparisons between LT and non‐LT patients should be considered exploratory. Although no statistically significant differences in CACS progression were observed between groups, the limited number of controls reduces the statistical power to detect modest but clinically meaningful differences and increases the risk of a Type II error. Therefore, the absence of statistically significant differences should not be interpreted as evidence of equivalence between groups, and firm conclusions regarding the independent effect of LT on CACS progression cannot be established. Consequently, these findings should be interpreted with caution and validated in larger multicenter cohorts with adequate statistical power to confirm the observed results. Second, selection bias may have influenced the results. On the one hand, patients underwent CACS_bl_ per clinical protocol based on the presence of CVR factors, so patients with lower probabilities of coronary calcifications were not included. On the other hand, the exclusion of patients who died, underwent coronary revascularization or other major cardiac interventions, or were unavailable for follow‐up may have introduced survivor bias. Individuals experiencing adverse cardiovascular outcomes during follow‐up may represent a higher‐risk subgroup with potentially more rapid progression of coronary atherosclerosis. As a result, the observed rates of CACS progression may underestimate the true burden of atherosclerotic progression within the overall LT population. Third, although CACS is a well‐established marker of coronary atherosclerotic burden and CVR, it does not provide information on noncalcified plaque burden, plaque composition, or other features associated with plaque vulnerability. Consequently, the absence of coronary CT angiography data limits our ability to fully characterize atherosclerosis progression. Another limitation relates to the assessment of CVR factors and immunosuppressive exposure at 1 year after LT, whereas CACS progression was evaluated over an approximately 5‐year interval. A single time‐point assessment may not accurately reflect cumulative exposure to CVR factors, changes in metabolic status, or modifications in immunosuppressive therapy occurring throughout follow‐up. Accordingly, residual confounding related to temporal variations in these factors cannot be excluded. The subgroup analyses, related to diabetes status, should also be interpreted with caution as they involved small sample sizes and were not powered to detect subgroup‐specific effects. Finally, differences in the intensity of cardiovascular surveillance between cohorts should also be considered when interpreting the results. LT recipients were managed within a structured multidisciplinary follow‐up program, which included systematic assessment and treatment of CVR factors. In contrast, individuals in the non‐LT cohort received routine clinical care. This difference in monitoring and risk‐factor management may have influenced cardiovascular outcomes and potentially attenuated differences in atherosclerosis progression between groups. In this regard, post‐LT patients at our center were closely monitored for CVR factors by a multidisciplinary team of specialists. This structured follow‐up may have allowed earlier identification and management of CVR factors than would occur in centers with different clinical pathways, resources, or follow‐up strategies. Therefore, our findings may not be fully generalizable to other centers where post‐LT cardiovascular monitoring is less intensive or not routinely coordinated by a dedicated team.

In summary, over a 5‐year period, CACS progression was not faster in a cohort of LT recipients with optimized control of CVR factors and standard/low CET than in patients with cirrhosis. CACS_bl_ and kidney dysfunction at 12 months after LT independently predicted progression of CACS after LT, but we did not find immunosuppression‐related or CVR variables associated with the progression of CACS after LT. This may indicate that these variables may affect progression of coronary atherosclerosis through their effects on kidney function. Prospective, multicenter studies may investigate whether interventions aiming at preserving kidney function after LT contribute to decelerate the progression of atherosclerosis in this population.

**Nomenclature**
AU: Agatston units
BMI: body mass index
BP: blood pressure
CACS: coronary artery calcium score
CET: cumulative exposure to tacrolimus
CT: computed tomography
CVD: cardiovascular disease
CVE: cardiovascular events
CVR: cardiovascular risk
DM: diabetes mellitus
ECG: electrocardiography
eGFR: estimated glomerular filtration rate
HbA1C: glycosylated hemoglobin
IQR: interquartile range
LDL: low density lipoprotein
LT: liver transplantation
SLD: steatotic liver disease

## Author Contributions

Giulia Pagano: performance of research, data analysis, and writing of the paper; Judit Mestres‐Martí: performance of research and critical review of the paper; Cautar El Maimouni: critical review of the paper; Clara Viñals: critical review of the paper; Jose T. Ortiz‐Pérez: critical review of the paper; Pablo Ruiz: critical review of the paper; Jordi Colmenero: critical review of the paper; Emilio Ortega: critical review of the paper; Gonzalo Crespo: study design, performance of research, data analysis, and writing of the paper.

## Funding

The authors have nothing to report.

## Conflicts of Interest

The authors declare no conflicts of interest.

## Data Availability

The data that support the findings of this study are available on request from the corresponding author. The data are not publicly available due to privacy or ethical restrictions.
